# Alterations in the Placenta Following Vaccination and Infection with SARS-CoV-2 During Pregnancy

**DOI:** 10.3390/ijms27125473

**Published:** 2026-06-17

**Authors:** Nils Hoymann, Laura Scholz, Suzan Alboradi, Valeriia Grabar, Gina Marie Uehre, Jakob Tong Khuankhunsathid, Eliane Tabea Taube, George Toth, József Mészáros, Paolo Gennari, Svetlana Tchaikovski, Atanas Ignatov, Mandy Busse

**Affiliations:** 1 Experimental Obstetrics and Gynecology, Medical Faculty, Otto-von-Guericke University, 39108 Magdeburg, Germanygina.uehre@med.ovgu.de (G.M.U.); 2 University Hospital for Obstetrics and Gynecology, Medical Faculty, Otto-von-Guericke University, 39108 Magdeburg, Germany; valeriia.grabar@med.ovgu.de (V.G.); jozsef.meszaros@med.ovgu.de (J.M.); paolo.gennari@med.ovgu.de (P.G.); svetlana.tchaikovski@uk-brandenburg.de (S.T.); atanas.ignatov@med.ovgu.de (A.I.); 3 Institute of Pathology, University Hospital Magdeburg, 39120 Magdeburg, Germany; jakob.khuankhunsathid@st.ovgu.de (J.T.K.); eliane.taube@med.ovgu.de (E.T.T.); 4 University Clinic for Obstetrics and Gynecology, Medical Faculty, Brandenburg Medical School Theodor Fontane, 14770 Brandenburg an der Havel, Germany

**Keywords:** SARS-CoV-2, placenta, vaccination, inflammation

## Abstract

Despite the years that have passed since the pandemic, data regarding the effects of mild SARS-CoV-2 infection and vaccination during pregnancy remain limited. The current study investigated the expression of molecules that may be involved in the placental immune response using real-time PCR and Western blot analysis in a well-characterized cohort of 118 placentas collected between the 37th and 40th week of gestation. Secreted mediators were assessed in the supernatant of placental cell cultures, and histological examinations of the placental tissue were performed. Significant differences in the expression levels of *S100B*, *IL6,* and *CCL5* were observed in control versus vaccinated and previously infected women, as determined by PCR. Acute SARS-CoV-2 infection decreased the expression of p38 MAPK and Bcl-2 compared to control patients. The secretion of G-CSF, IFN-α2, IL-2, and CXCL8 (IL-8) increased in women who were infected during pregnancy and/or vaccinated. However, histological analysis revealed only minor differences between the groups. In conclusion, SARS-CoV-2 infection or vaccination during pregnancy induced a measurable placental immune response that remained below the threshold of histologically detectable tissue injury.

## 1. Introduction

Notwithstanding the end of the 2019 Coronavirus Disease (COVID-19) pandemic, the consequences of the disease remain an ongoing global challenge of major social importance. The COVID-19 pandemic was attributed to the global spread of the pathogen severe acute respiratory syndrome coronavirus 2 (SARS-CoV-2). The virus initially binds to epithelial cells in the oral and nasal cavities before migrating further into the conducting airways of the respiratory tract [1]. Angiotensin-converting enzyme 2 (ACE2) and transmembrane serine protease 2 (TMPRSS2) have been identified as entry factors for SARS-CoV-2 infection [2] and are highly expressed in the upper respiratory tract, together with targets important for innate immunity [3]. Following the infection of host cells, SARS-CoV-2 viral single-stranded RNA (ssRNA) and its replication intermediate, double-stranded RNA (dsRNA) are detected by immune cells via Toll-like receptors (TLRs) 7 and 8, as well as TLR3 [4]. However, TLRs 2 and 4 have also been identified as receptors for SARS-CoV-2 [5,6]. TLRs are pattern-recognition receptors that sense common patterns shared by various pathogens, known as pathogen-associated molecular patterns (PAMPs), including ssRNA and dsRNA. Myeloid differentiation factor 88 (MyD88) is a downstream adaptor protein for the majority of TLRs and is essential for signaling transduction following TLR engagement [7]. MyD88 signal transduction activates the nuclear factor ‘κ light-chain-enhancer’ of activated B-cells (NF-κB) signaling pathway, leading to a cascade of inflammatory responses. TLRs also sense damage-associated molecular patterns (DAMPs). S100B and high-mobility group box 1 (HMGB1) are DAMP molecules that serve as danger signals to activate the immune system in tissue damage or cellular stress. S100B and HMGB1 bind to receptors such as the receptor for advanced glycation end products (RAGE) and TLRs, thus inducing a robust inflammatory response [8,9]. SARS-CoV-2 infection induces the secretion of HMGB1 and S100B, which might serve as marker for COVID-19 severity [10,11]. Furthermore, RAGE has been demonstrated to facilitate the infection of monocytes by the SARS-CoV-2 N protein, thereby activating NF-κB, which in turn leads to the production of key pro-inflammatory cytokines. It is evident that RAGE engagement has a significant impact on the severity of SARS-CoV-2 infection [12,13].

Although no increased susceptibility to SARS-CoV-2 infection during pregnancy was reported [14], pregnant women are at increased risk of developing severe COVID-19 [15]. Furthermore, gestational infection with SARS-CoV-2 has been associated with stillbirth, preterm birth, and neonatal intensive care unit (ICU) admission, risks that can be reduced by vaccination during pregnancy [16]. Immunological adaptations required to maintain feto-maternal tolerance may contribute to the increased risk of severe COVID-19 during pregnancy. This tolerance depends on a tightly regulated balance of inflammatory and anti-inflammatory immune cells [17]. At the same time, the maternal immune system must protect both the mother and fetus against infection. Although intrauterine transmission of SARS-CoV-2 is rare, soluble mediators, such as cytokines and chemokines, and maternal protective antibodies, can cross the placenta to the fetus [18]. SARS-CoV-2 infection has also been associated with placentitis, characterized by histiocytic intervillositis, perivillous fibrin deposition, and necrosis of villous trophoblast [19,20].

Several molecules involved in the immune response to SARS-CoV-2 also play important roles in physiological pregnancy and pregnancy-associated complications. The human placenta expresses the main SARS-CoV-2 entry factors, ACE2 and TMPRSS2 [21], as well as all TLRs (1–10) [22]. ACE2-expressing maternal and fetal cells within the placenta can bind to SARS-CoV-2, resulting in decreased placental ACE2 expression during acute infection [23]. RAGE is a key mediator of inflammatory processes during pregnancy, contributing to fetal membrane weakening and becoming activated in the uterus, placenta, and cervix toward the end of gestation [24]. Its ligand HMGB1 is involved in physiological inflammatory processes, including implantation and the initiation of labor. However, HMGB1 also contributes to the pathogenesis of inflammation-associated pregnancy complications, such as preterm birth [25].

Therefore, the engagement of receptors and the subsequent activation of signaling pathways may be influenced by SARS-CoV-2 infection, vaccination, or gestational age. In order to differentiate placental alterations associated with infection and/or vaccination from physiological pregnancy-related changes, we performed a comprehensive analysis of inflammatory and signaling molecules in placentae obtained after the 37th week of gestation and compared them with those from non-vaccinated, non-infected women.

## 2. Results

### 2.1. Patient Cohort

The study cohort included a total of 118 women distributed as follows: 14 unvaccinated and uninfected pregnant patients (controls), 21 vaccinated but uninfected women, 22 unvaccinated women with documented SARS-CoV-2 infection during pregnancy, 48 women who were both vaccinated and infected, and 13 women with documented acute infection at the time of delivery (Table 1). The groups differed in gestational age and newborn body length. In comparison to the controls, delivery complications occurred more frequently among women who were both vaccinated and previously infected (*p =* 0.03). However, this difference was not statistically significant in the other groups. As demonstrated in Table 1, a number of significant alterations were identified between the groups with respect to the trimester of SARS-CoV-2 infection, the SARS-CoV-2 ct value at birth, the number of vaccinations administered, and the trimester of the most recent vaccination.

### 2.2. Alteration in the Gene Expression of Immune Factors Potentially Involved in SARS-CoV-2 and Parturition

In order to investigate alterations at the maternal–fetal interface following SARS-CoV-2 infection or vaccination during pregnancy, the placental expression of several genes associated with immune cell signaling and response was analyzed. The placental expression of *HMBG1*, *RAGE,* and *MYD88* remained unchanged in response to SARS-CoV-2 infection or vaccination (Table 2). Compared with controls, *S100B* expression was reduced in both vaccinated and infected women, as well as in acutely infected patients. The placental expression of *IL6* was higher in controls and vaccinated-only patients than in the infected groups (infected only, infected and vaccinated, and acutely infected). In addition, *CCL5* and *IL17A* expression levels were lower in vaccinated and infected women compared with unvaccinated infected patients. *S100B* expression correlated positively with *IL-17A* expression in the control group (Spearman r = 0.8605; *p =* 0.0014), negatively with *IL6* expression in the infected-only group (Spearman r = −0.5292; *p =* 0.0239), and positively with *CCL5* expression in the infected and vaccinated group (Spearman r = 0.3474; *p =* 0.0326). Neither previous nor acute SARS-CoV-2 infection, nor vaccination, affected placental expression of *IL1B*, *CXCR4*, *FAS,* or *MMP9*. The expression of *FASL*, *TNFA*, *IFNG,* and *IL10* was only rarely detected in placental tissue.

### 2.3. SARS-CoV-2 Infection Altered the Placental Expression of Pro- and Anti-Apoptotic Proteins

**Figure 1:**
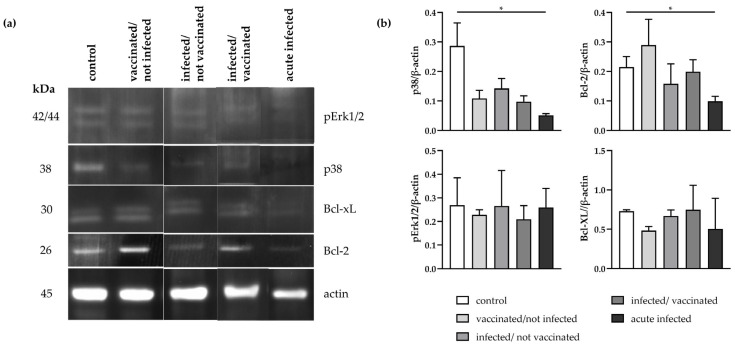
(**a**) Placental expression of pro- and anti-apoptotic proteins. The figure illustrates the expression levels of phosphorylated and total Erk1/2 (p42/p44), p38, Bcl-xL, Bcl-2, and β-actin in the control group and in patients vaccinated but not infected, infected but not vaccinated, both infected and vaccinated, and acutely infected with SARS-CoV-2 during delivery. As illustrated in Figure (**b**), the results of the analysis of the band heights are presented, with the use of Syngene GeneTools Western blot analysis software. * *p* < 0.05.

Placental expression of pro- and anti-apoptotic proteins was measured by Western blot. Total p38 MAPK expression was at the highest levels in control placentae. In acutely infected patients, p38 MAPK expression was significantly lower (Figure 1a,b), and pp38 was not detected in any of the samples. Expression of total Erk1/2 and phosphorylated Erk1/2 (pErk1/2, Figure 1a,b) was not affected by infection or vaccination.

Expression of the anti-apoptotic protein Bcl-2 was elevated in women infected during pregnancy and was significantly reduced in acutely infected patients compared to control patients (Figure 1a,b). Placental expression of Bcl-xL was slightly decreased in the vaccinated-only patients compared to control women.

### 2.4. SARS-CoV-2 Infection Influenced the Placental Secretion of Immune Mediators

**Figure 2:**
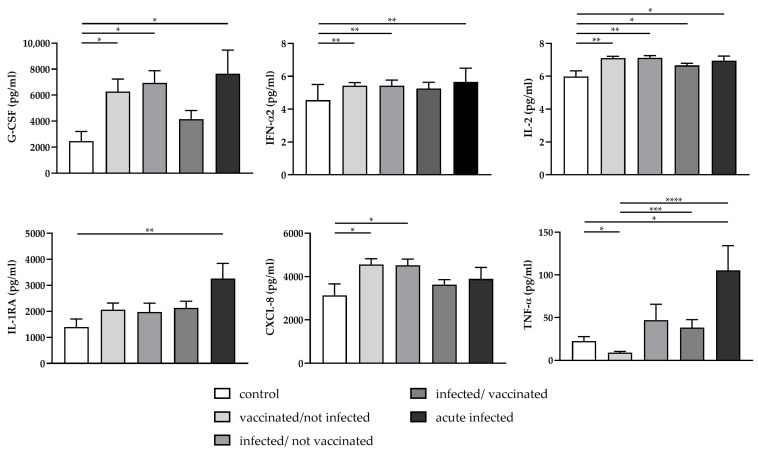
Placental secretion of SARS-CoV-2-associated immune mediators. The figure illustrates the secretion levels of G-CSF. IFN-α2, IL-2, IL-1RA, CXCL-8, and TNF-α (in pg/mL) in the control group and in patients vaccinated/not infected, infected/not vaccinated, both infected and vaccinated, and acutely infected with SARS-CoV-2 during delivery. * *p* < 0.05, ** *p* < 0.01, *** *p* < 0.001, **** *p* < 0.0001.

Exposure to SARS-CoV-2 antigens, either through infection or through vaccination, altered the placental secretion of G-CSF (*p =* 0.0072), IFN-α2 (*p =* 0.0012), CXCL8 (*p =* 0.0298), IL-2 (*p =* 0.0028), IL-1RA (*p =* 0.0548), and TNF-α (*p* <0.0001; Figure 2). Compared to the control group, acute SARS-CoV-2 infection resulted in increased secretion of MCP-1 (*p =* 0.0123), G-CSF (*p =* 0.0313), IFN-α2 (*p =* 0.0088), IL-2 (*p =* 0.0394), IL-1RA (*p =* 0.0056), and TNF-α (*p =* 0.0374) in placentae. Other mediators potentially involved in the SARS-CoV-2-induced cytokine storm were not altered by infection or vaccination (Table S2).

### 2.5. SARS-CoV-2 Infection During Pregnancy Is Associated with Only Minor Histopathological Changes in Placenta

**Figure 3:**
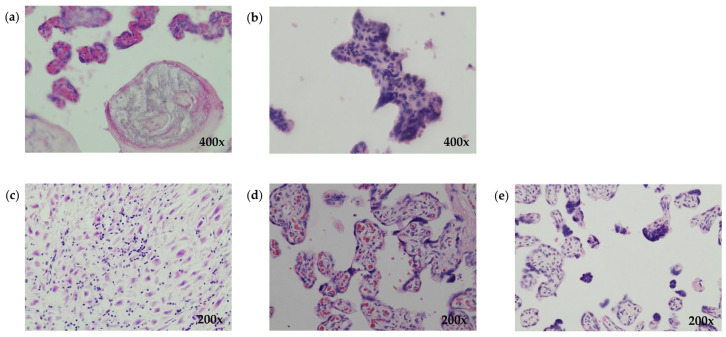
Representative images of histological findings of placentae. The placentae were subjected to H&E staining and then examined by experienced pathologists. The following histopathological features are demonstrated: (**a**) calcification, (**b**) avascular villus, (**c**) lymphocytes, (**d**) syncytial bridge, (**e**) free syncytial nodule.

Histological examination of placentae of women who underwent SARS-CoV-2 vaccination, mild SARS-CoV-2 infection, or the combination of both during pregnancy remains scarce and contradictory. A comprehensive analysis of histopathological features of the placenta, including calcifications, lymphocyte infiltration, avascular villi, and syncytial nodes, was conducted. Representative images illustrating these features are presented in Figure 3.

No significant differences were identified between placentae across the groups (Table 3).

## 3. Discussion

In our study, we detected differences in the expression of several signaling- and apoptosis-associated molecules (p38, Bcl-2) and immune mediators (including S100B, IL-6, IL-17A, CCL5) in the placentae of women who delivered following vaccination and/or SARS-CoV-2 infection during pregnancy. Furthermore, placental secretion of mediators (G-CSF, IFN-α2, IL-2, CXCL-8, and TNF-α) differed between the patient groups. However, these alterations appeared to have no effect on placental histology. Our data thus support the concept of functional reprogramming of the placenta following mild infection and/or vaccination, as opposed to that of overt tissue damage.

Firstly, it is imperative to acknowledge that parturition at term is typified by an inflammatory response in the feto-maternal tissues, such as the placenta. A number of pro-inflammatory mediators, including IL-6, IL-8, IL-1β, COX-2, PGE-2, TNF-α, and hCAP18, have been identified during term labor [26]. Furthermore, it appears plausible that maternal infection may influence the placenta’s immune response to inflammation. The objective of this study was to determine whether a prior vaccination or SARS-CoV-2 infection resulted in an elevated inflammatory immune response or whether anti-inflammatory counter-regulation occurred. SARS-CoV-2 can bind to TLRs, thereby activating MyD88-dependent pathways that subsequently induce NF-κB signaling and the expression of pro-inflammatory mediators such as TNF-α, IL-1β, IL-6, IL-8, IL-12, IL-17A, iNOS, and IFN-γ [7]. Both our findings and those of other groups indicate that MyD88 is essential for the development of physiological pregnancy [27,28,29]. Nevertheless, we did not reveal any differences in *MYD88* expression among the investigated groups. It has also been shown that SARS-CoV-2 might bind directly to RAGE or indirectly via HMGB1 upon infection of several cell types. We could not detect alterations in placental *RAGE* or *HMGB1* expression across the groups, including patients with acute infection.

Nonetheless, we observed significant alterations in placental *S100B* expression. The biological effects of S100B depend on three major factors: its concentration, the target cell type, and the surrounding microenvironment. Data on the expression and secretion of S100B by the placenta remain limited. Previously, we demonstrated increased placental S100B secretion in preterm delivery and preeclampsia, both conditions associated with inflammation [30]. However, the directionality of the relationship between inflammatory cytokines (TNF-α, IL-6) and S100B remains unclear. It is possible that S100B is induced by inflammatory cytokines, or conversely, that inflammatory cytokines are induced by S100B [31,32,33]. In the present study, acute SARS-CoV-2 infection was associated with elevated placental TNF-α secretion, while *S100B* expression levels remained low. This finding might suggest that TNF-α may function as an inducer of placental S100B expression following SARS-CoV-2 infection, a possibility that should be investigated further in subsequent experiments.

In accordance with prior observations, we detected elevated placental *CCL5* expression in women positive for SARS-CoV-2 infection [34]. Furthermore, placental *IL17A* expression was increased in acutely infected patients. IL-17 has been identified as a potential factor in the establishment of pregnancy and angiogenesis, but has also been associated with pregnancy complications such as preeclampsia and infection-induced preterm delivery [35,36,37]. Genes such as *IL6* and *CXCR8* have been shown to be upregulated by SARS-CoV-2, possibly contributing to the activation of the IL-17 signaling pathway [38] in a few organs, whereas data regarding placental tissue remain limited. We observed elevated levels of placental *IL-17A* in women with acute SARS-CoV-2 infection or those who had previously been vaccinated and infected. Conversely, *IL6* expression was not increased in any SARS-CoV-2-infected groups, including acutely infected patients. Similarly, previous studies demonstrated no significant differences in placental *IL6* expression between symptomatic SARS-CoV-2 patients and controls, and even higher levels of *MCP1, IL6, IFNG*, *IL8,* and *IL1B* in the latter group compared to asymptomatic patients [39,40,41]. Furthermore, placental expression of type I IFNs and inflammatory cytokines (IFNB, IL6, IL1B) appears to decrease with increasing time between the initial SARS-CoV-2 infection and delivery [42].

We did not detect differences in the placental expression of *S100B*, *CCL5,* and *IL17A* between the control, infected-only, and vaccinated-only groups. This finding is consistent with the conclusions of another study, which demonstrated that vaccination did not result in placental inflammation when compared to the control group [43]. Interestingly, we found that women who were both vaccinated and infected with SARS-CoV-2 during pregnancy exhibited reduced placental expression of *S100B*, *IL6*, *CCL5,* and *IL17A,* compared with the control group. These observations require further experimentation to identify the underlying mechanisms.

NF-κB, Erk, IL-2, IL-6, and TNF-α signaling pathways are likely key contributors to the inflammatory response during the early stages of SARS-CoV-2 infection, including in the placenta [44]. Furthermore, RAGE engagement by S100B can induce the expression of inflammatory molecules such as IL-6, TNF-α, CCL5, iNOS, and IL-1β via NF-κ activation [45,46]. Additionally, the interaction between S100B and RAGE can also modulate apoptosis by altering Bcl-2 expression [47,48]. p38 MAPK is a major stress-activated kinase that regulates the production of inflammatory cytokines and immune signaling. In reproductive tissues, it is activated by oxidative stress and pro-inflammatory cytokines, contributing to the production of cytokines and chemokines associated with pregnancy and parturition [49]. It was shown that p38 was phosphorylated following infection of a human lung cell line with SARS-CoV-2 [50]. We found that the expression of p38 was significantly reduced in the placentae of patients with acute infections, and the expression of pp38 was undetectable. The p38 MAPK pathway has been identified as a pivotal stress- and inflammatory-signaling pathway. Reduced expression or activation may indicate an altered cellular response to viral exposure. However, information on the contribution of p38 signaling to SARS-CoV-2 infection and vaccination is very limited. SARS-CoV-2 induces Erk1/2 phosphorylation in a number of cell lines [51], but this was not detectable in the placentae of acutely infected women. However, it is known that the p38 and Erk signaling pathways interact with each other, particularly during parturition. Therefore, the specific role of reduced placental expression of p38 in women with acute infections, as well as potential compensatory mechanisms, should be investigated in greater detail.

Bcl-2 and Bcl-xL are members of the BCL2 family exhibiting anti-apoptotic properties. Placental Bcl-2 expression is increased in pregnancy and unaffected by the mode of delivery [52,53]. In our study, acutely infected women had a very low expression of *Bcl-2* in their placentae. A reduction in Bcl-2 expression has been shown to result in an imbalance, thereby increasing susceptibility to apoptosis. This may lead to enhanced apoptosis of placental cells, as Bcl-2 inhibits trophoblast apoptosis [54]. Furthermore, reduced Bcl-2 and increased apoptosis were linked to SARS-CoV-2-associated placentitis [55], but we did not observe placentitis in our study cohort. Nevertheless, such a molecular shift does not necessarily result in visible changes at a histological level, particularly since mild infections or compensatory mechanisms may be present. Although changes in apoptotic signaling pathways were detectable, they were apparently insufficient to cause structural damage to the placental tissue that could be identified using conventional histopathology techniques.

With the exception of the control group, placental IFN-α2 secretion was increased across all patient groups. This observation is consistent with other studies demonstrating that type I interferons are essential for anti-viral immune response and can be induced both by SARS-CoV-2 mRNA vaccines and maternal SARS-CoV-2 infection [56,57]. Additionally, placental secretion of G-CSF, IL-2, and CXCL8 was lowest in the control group. Previous studies demonstrated alterations of these immune mediators during SARS-CoV-2 infection [58,59]. Our observation of similar changes after vaccination suggests that exposure to the spike protein alone is sufficient to induce a placental immune reaction comparable to that observed during infection. Plasma G-CSF levels were reported to increase in moderate and severe SARS-CoV-2 infection [60]. Beyond its established role in neutrophil maturation and chemotaxis [61], G-CSF is expressed by the placenta throughout pregnancy, where it contributes to IL-1- and TNF-α-mediated immune regulation, angiogenesis, cell migration, and inhibition of apoptosis [62]. Increased placental G-CSF has been shown in intra-amniotic infection [63], suggesting that a similar inflammatory mechanism may also occur during acute SARS-CoV-2 infection or persist following maternal infection.

IL-2 has also been identified as an “immune signature” of SARS-CoV-2 infection [64]. In the placenta, IL-2 is primarily secreted by decidual immune cells following TLR engagement and supports the function of the NK and CD8+ T cells, particularly through IFNγ production [65,66]. Although this mechanism has not yet been directly observed in maternal SARS-CoV-2 infection, it represents a plausible component of antiviral response. Among all measured mediators, IL-6 and CXCL8/IL-8 showed the highest concentrations in placental culture supernatant. This is not surprising, since both mediators play a key role in human parturition and are increased in healthy term placentae during spontaneous labor. However, these biomarkers are even further enhanced in pregnancy complications such as preeclampsia and maternal infections [67,68]. During labor, IL-6 contributes to the induction and amplification of CXCL8 expression, which, in turn, promotes neutrophil chemoattraction, degranulation, and MMP release. Although IL-6 secretion did not differ between the groups, we observed significant differences in placental CXCL8 release. These findings suggest that inflammatory processes leading to the upregulation of IFN-α2, G-CSF, IL-2, and CXCL8 are amplified following maternal exposure to the SARS-CoV-2 spike protein, irrespective of whether exposure occurs through vaccination or infection. However, further studies are required to elucidate the precise mechanisms underlying these placental immune responses.

Data regarding SARS-CoV-2-induced placental pathology remain inconsistent and, in some cases, contradictory. Several studies have reported maternal and fetal vascular malperfusion, villitis, funisitis, chorioamnionitis, and chronic histiocytic intervillositis in placentae from infected women [69,70,71]. Watkins et al. [72] introduced the term “SARS-CoV-2 placentitis” to describe the typical triad of syncytiotrophoblast necrosis, increased perivillous fibrin deposition, and chronic histiocytic intervillositis in infected placentae, accompanied by infiltration of inflammatory cells, including lymphocytes [73,74]. Several pathophysiological mechanisms linked to SARS-CoV-2 infection, including endothelial dysfunction, inflammation, and thrombogenic alterations, overlap with those observed in pregnancy complications such as preeclampsia. Since women with PE, IUGR, or preterm delivery were excluded from our study, the absence of these pathological findings was not unexpected.

SARS-CoV-2 may induce hypercoagulability and inflammation even in the absence of overt histopathological changes, manifesting as intervillous thrombosis and fibrin deposition [75,76,77]. However, these alterations were not observed in our study cohort, including women with acute infection, consistent with previous reports showing that SARS-CoV-2 infection during pregnancy, irrespective of disease severity or gestational age at infection, does not necessarily result in significant placental histopathological changes [78].

In women with acute SARS-CoV-2 infection, the present findings suggest that the placenta is not necessarily undergoing destructive changes; rather, the placenta may be demonstrating an active antiviral and immunological response. Furthermore, our clinical data, including normal birth weight, normal APGAR scores, normal umbilical cord pH levels, no increase in fetal abnormalities, and no increased morbidity, do not suggest significant placental pathology. In the event of structural damage to the placenta, it is generally expected that there would be a demonstrable trend in these parameters.

Much less is known about the impact of SARS-CoV-2 vaccination on the placenta. However, available evidence, supported by our findings, indicates that vaccination during pregnancy is not associated with adverse histopathological placental changes [79,80].

Our study has some limitations. The present study detected changes in the expression of certain markers via PCR. It is acknowledged that the detection of marker expression via PCR does not necessarily reflect protein expression. We determined the presence of variations in gestational age and delivery complications among the study groups, and it is possible that these factors influenced the results, which we did not further address. 

Overall, the increased secretion of IFN-α2, IL-2, TNF-α, G-CSF, and IL-1RA in placentas from acutely infected women suggests activation of both antiviral and inflammatory signaling pathways. However, the concomitant increase in regulatory mediators such as IL-1RA and the absence of major histopathological abnormalities indicate that these responses may represent a controlled adaptive reaction rather than overt placental injury. Consequently, the observed molecular alterations are indicative of functional immune modulation within the placenta, with no significant structural damage observed.

## 4. Materials and Methods

### 4.1. Study Cohort and Sampling

The study was approved by the Ethics Committee of the Otto-von-Guericke University (Medical Faculty, EK19/22). Prior to participation, all patients were provided with detailed information regarding the study objectives and were asked to provide their written consent. Inclusion criteria included reaching the 37th week of gestation, completing the questionnaire, and consenting to the study. Exclusion criteria included experiencing pregnancy complications and other infections during pregnancy. Immediately after delivery, placentae were transported to the laboratory. Following a thorough washing with phosphate-buffered saline (PBS), villous placental tissue was snap-frozen. For explant culture, villous placental tissue was carefully dissected into 500 mg pieces, transferred into 24-well plates, and cultured for 24 h in RPMI 1640 supplemented with 3% charcoal-treated fetal bovine serum and 1% penicillin/streptomycin, as previously described [30]. Subsequently, culture supernatants were harvested, centrifuged (3000 rpm, 10 min, RT), snap-frozen, and stored at −80 °C until further analysis.

### 4.2. Cytokine Detection in Placental Explants

Cytokines and chemokines were measured using the LEGENDplex™ COVID-19 Cytokine Storm Panel 1 (13-plex; Biolegend, San Diego, CA, USA) according to the supplier’s recommendations. Measurements were performed using an Attune NxT flow cytometer (Thermo Fisher Scientific, Waltham, MA, USA) and analyzed with FlowJo software v.10.10.1 (FlowJo LLC, Ashland, OR, USA).

### 4.3. RNA Isolation and Real-Time Reverse Transcriptase Polymerase Chain Reaction (RT-PCR)

Total RNA was isolated from frozen tissues using TRIzol (Ambion, Thermo Fisher Scientific, Dreieich, Germany) as previously described [28]. Briefly, 1 g of placental tissue was homogenized in 1 mL of TRIzol, followed by the addition of chloroform and isopropanol. The tissue was then washed with ethanol, and the RNA pellet was resuspended in RNAse-free dH2O at a concentration of 1 µg/µL.

RT-PCR amplifications were performed using an iCycler (iQ5, BioRad, Feldkirchen, Germany). The primers for *RAGE*, *S100B*, *HMGB1*, *MYD88*, *INOS*, *FAS*, *FASL IL1B*, *IL6*, *IL10*, *IL17A*, *TNFA*, *IFNG*, *CCL5*, *CXCR4*, *MMP9,* and *ACTB* are listed in Table S1. All experiments were conducted in duplicate, with an initial denaturation step at 95 °C for 5 min, followed by 40 cycles of denaturation for 45 s at 95 °C and annealing for 60 s at 60 °C for amplification.

### 4.4. Western Blot Analysis

Western blot analysis was performed as previously described [81]. Briefly, the placental tissue was sonicated in a lysis buffer and incubated on ice for 20 min, followed by centrifugation at 12,000 rpm for 30 min at 4 °C. Protein concentrations were determined by the Bradford assay (Biorad, Feldkirchen, Germany). Protein samples were separated by SDS-PAGE and transferred for Western blot analysis. Membranes were blocked with 5% BSA/TBS and incubated overnight at 4 °C under constant rotation with the following primary antibodies: phospho-p38, total p38, phospho-Erk1/2, total Erk1/2, Bcl-xL, Bcl-2 (all from Cell Signaling Technology, Danvers, MA, USA), and β-actin (Santa Cruz Biotechnology, Dallas, TX, USA). After washing with TBS/Tween 20, membranes were incubated with an HRP-conjugated goat anti-rabbit secondary antibody (Cell Signaling Technology, Danvers, MA, USA). Signal detection was performed using an HRP substrate (Merck Millipore, Darmstadt, Germany). The automatic detection and quantification of band heights and volumes were performed using Syngene GeneTools (version 4.01.09), a software program designed for the analysis of Western blots.

### 4.5. Histology

Immediately after delivery, placental tissue was dissected into approximately 1 cm^3^ fragments and repeatedly washed with PBS. Samples were fixed in 4% (*w*/*v*) paraformaldehyde (PFA) with 0.1 M sucrose (pH 7.4) for 6 h, dehydrated with 100% ethanol and xylene, embedded in paraffin, and sectioned at 4 µm thickness. For deparaffinization, sections were incubated twice in xylene for 10 min each, followed by rehydration through a descending series of ethanol solutions (100%, 95%, 75%) for 5 min each, and rinsed in distilled water. Sections were stained with hematoxylin for 2–5 min, washed in lukewarm tap water for 5 min, rinsed in distilled water, and counterstained with eosin for approximately 20 s. Finally, sections were rinsed with distilled water, clarified twice in xylene for 5 min each, and mounted with Histotek mounting medium.

### 4.6. Data Analysis and Statistics

Statistical analysis was performed using GraphPad Prism 8.0 software. The normality of data distribution was determined using the Shapiro–Wilk test. The analysis of the data was performed using either a one-way analysis of variance (ANOVA) followed by Dunnett’s multiple comparisons test or a Kruskal–Wallis test followed by Dunn’s multiple comparisons test. The measurement of contingency was conducted using a two-sided Fisher’s exact test. Significance was defined as follows: * *p* < 0.05, ** *p* < 0.01, *** *p* < 0.001, **** *p* < 0.0001.

## Figures and Tables

**Table 1:** Clinical Characteristics of the study cohort.

Characteristics	Control (N = 14)	Vaccinated/Not Infected (N = 21)	Infected/Not Vaccinated (N = 22)	Infected/Vaccinated (N = 48)	Acute Infected (N = 13)	*p*
***maternal***						
age (years)	31.3 ± 6.7	29.2 ± 4.7	31.1 ± 6.4	32.2 ± 6.3	31.9 ± 4.6	0.4031
gestational age (weeks)	38.0 ± 1.8	39.5 ± 1.3	38.1 ± 1.4	38.6 ± 1.6	39.3 ± 1.3	0.0134
birth mode						
vaginal birth	5 (36%)	13 (62%)	11 (50%)	16 (33%)	6 (46%)	0.2245
cesarean section	9 (64%)	8 (38%)	11 (50%)	32 (67%)	7 (54%)	
gravida	32.5 ± 1.5	2.3 ± 1.8	3.2 ± 2.1	2.9 ± 1.9	2.5 ± 1.6	0.3469
para	2.0 ± 1.4	1.8 ± 1.0	2.3 ± 1.3	2.1 ± 1.2	2.1 ± 1.2	0.7085
blood loss (ml)	412 ± 108	345 ± 133	380 ± 175	435 ± 165	489 ± 324	0.1893
delivery complications						
yes	4 (29%)	13 (62%)	9 (41%)	31 (65%)	8 (62%)	0.0870
no	10 (71%)	8 (38%)	13 (59%)	17 (35%)	5 (38%)	
***neonatal***						
ultrasound abnormalities						
yes	2 (15%)	2 (10%)	3 (15%)	6 (13%)	3 (30%)	0.6000
no	11 (85%)	19 (90%)	17 (85%)	40 (87%)	7 (70%)	
birth weight (g)	3161 ± 555	3371 ± 458	3224 ± 496	3352 ± 661	3574 ± 629	0.3691
body length (cm)	49.4 ± 2.3	51.4 ± 2.3	50.6 ± 2.3	50.8 ± 3.8	52.6 ± 2.3	0.0239
APGAR (1 min)	9.3 ± 0.6	9.0 ± 1.2	9.0 ± 0.9	8.7 ± 1.4	9.2 ± 0.4	0.3424
APGAR (5 min)	9.8 ± 0.6	9.5 ± 1.2	9.6 ± 0.7	9.5 ± 0.9	9.9 ± 0.4	0.6016
APGAR (10 min)	10.0 ± 0.0	9.9 ± 0.4	9.9 ± 0.5	9.8 ± 0.5	10.0 ± 0.0	0.2052
CB pH	7.3 ± 0.07	7.3 ± 0.08	7.3 ± 0.06	7.3 ± 0.08	7.3 ± 0.07	0.4412
CB base excess (mmol/L)	−2.1 ± 2.8	−2.0 ± 3.2	−0.5 ± 1.9	−1.8 ± 3.5	−3.1 ± 3.2	0.1991
***SARS-CoV-2***						
infection (trimester)	0	0	2.4 ± 1.4	2.8 ± 1.4	4 ± 0	<0.0001
ct value at birth	0	0	0	0	28.2 ± 8.6	<0.0001
last vaccination (trimester)	0	3.3 ± 1.6	0	3.5 ± 1.7	2.4 ± 2.3	<0.0001
vaccination (number)	0	2.3 ± 0.7	0	2.3 ± 0.8	1.8 ± 1.2	<0.0001

**Table 2:** Expression of genes in the placenta according to maternal vaccination and infection status.

	Control	Vaccinated/Not Infected	Infected/Not Vaccinated	Infected/Vaccinated	Acute Infected	*p*
*RAGE*	0.0010 ± 0.0011	0.0015 ± 0.0028	0.0028 ± 0.0036	0.0008 ± 0.0011	0.0014 ± 0.0021	0.4440
*HMGB1*	0.0760 ± 0.0487	0.1727 ± 0.1723	0.1238 ± 0.0942	0.0914 ± 0.0726	0.1133 ± 0.0991	0.6005
*S100B*	0.0007 ± 0.0008	0.0005 ± 0.0006	0.0012 ± 0.0017	0.0001 ± 0.0002	0.0001 ± 0.0002	0.0078
*MYD88*	0.0002 ± 0.0002	0.0002 ± 0.0003	0.0001 ± 0.0003	0.0001 ± 0.0002	0.0003 ± 0.0004	0.7400
*MMP9*	0.0021 ± 0.0033	0.0013 ± 0.0022	0.0020 ± 0.0023	0.0014 ± 0.0018	0.0043 ± 0.0063	0.5956
*IL1B*	0.0006 ± 0.0011	0.0014 ± 0.0017	0.0020 ± 0.0037	0.0008 ± 0.0009	0.0015 ± 0.0023	0.6460
*IL6*	0.0055 ± 0.0070	0.0079 ± 0.0084	0.0012 ± 0.0020	0.0021 ± 0.0021	0.0027 ± 0.0048	<0.0001
*IL17A*	0.0023 ± 0.0036	0.0037 ± 0.0076	0.0042 ± 0.0048	0.0016 ± 0.0022	0.0071 ± 0.0131	0.0586
*CCL5*	0.0012 ± 0.0018	0.0022 ± 0.0022	0.0022 ± 0.0018	0.0008 ± 0.0009	0.0019 ± 0.0028	0.0183
*CXCR4*	0.0142 ± 0.0148	0.0180 ± 0.0261	0.0211 ± 0.0158	0.0187 ± 0.0182	0.0174 ± 0.0123	0.4777
*FAS*	0.0017 ± 0.0037	0.0023 ± 0.0042	0.0019 ± 0.0043	0.0007 ± 0.0009	0.0003 ± 0.0005	0.4468

The mean ± SD values of the 2^−∆ct^ data and the results of the Kruskal–Wallis tests are shown.

**Table 3:** Histopathological placental features.

	Control	Vaccinated/Not Infected	Infected/ Not Vaccinated	Infected/Vaccinated	Acute Infected
**Calcifications**	2/10 (20%)	0/9	0/15	2/25 (8%)	3/13 (23.1%)
**Lymphocytes**	0/10	0/9	2/15 (13.3%)	3/25 (12%)	1/13 (7.7%)
**Avascular villi**	0/10	0/9	1/15 (6.7%)	1/25 (4%)	3/13 (23.1%)
**Syncytial nodes**	1/10 (10%)	0/9	2/15 (13.3%)	0/25	0/13

Data are presented as the number of cases/total number of subjects in each group, with percentages shown in parentheses. The differences were not significant (*p* > 0.5).

## Data Availability

The raw data supporting the conclusions of this article will be made available by the authors, without undue reservation.

## Supplementary Materials

The following supporting information can be downloaded at: https://www.mdpi.com/article/10.3390/ijms27125473/s1.

## Abbreviations

The following abbreviations are used in this manuscript:

RAGE	Receptor for Advanced Glycation Endproducts
HMGB1	High-Mobility Group Box 1
FASL	FAS ligand
CCL5	CC-chemokine ligand 5
Erk	extracellular signal-regulated kinases
Bcl-2	B-cell lymphoma 2
Bcl-xL	B-cell lymphoma-extra large
IL	Interleukin
TNF	Tumor necrosis factor
MMP9	Matrix metalloproteinase-9
CXCR4	C-X-C chemokine receptor type 4
IFN	Interferon
INOS	Inducible nitric oxide synthases
MYD88	Myeloid differentiation primary response 88
