# Supplementary material for: Alterations in the Placenta Following Vaccination and Infection with SARS-CoV-2 During Pregnancy
Source: Int J Mol Sci. 2026 Jun 17;27(12):5473. doi: 10.3390/ijms27125473 (PMC13299606; doi:10.3390/ijms27125473)
Supplement: Supplementary file 1 [file ijms-27-05473-s001.zip › ijms-4360918-supplementary.pdf]

Supplementary Materials

Table S1. Primer sequences.

Primer	Primer Sequence (5′→3′)
RAGE	fw ACT-ACC-GAG-TCC-GAG-TCT-ACC
S100B	rev GTA-GCT-TCC-CTC-AGA-CAC-ACA<br><br>fw CAT-TTC-TTA-GAG-GAA-ATC<br>rev ATG-TTC-AAA-GAA-CTC-GT
HMGB1	Fw TGA-GCT-CCA-TAG-AGA-CAG-CG<br>Rev GCA-GAC-ATG-GTC-TTC-CAC-CT
FAS	Fw CAA-GGG-ATT-GGA-ATT-GAG-GA
	Rev ACC-TGG-AGG-ACA-GGG-CTT-AT
FASL	Fw GGG-CTG-TGT-CTC-CTT-GTG-AT<br>Rev TGC-CAG-CTC-CTT-CTG-TAG-GT
CCL5	Fw TCA-TTG-CTA-CTG-CCC-TCT-GC<br>Rev TAC-TCC-TTG-ATG-TGG-GCA-CG
IL1B	Fw AGC-TAC-GAA-TCT-CCG-ACC-AC<br>Rev CGT-TAT-CCC-ATG-TGT-CGA-AGA-A
IL6	Fw ACT-CAC-CTC-TTC-AGA-ACG-AAT-TG<br>Rev CCA-TCT-TTG-GAA-GGT-TCA-GGT-TG
IL10	Fw CCG-AGA-TGC-CTT-CAG-CAG-AGT-G<br>Rev CAC-CTC-CTC-CAG-GTA-AAA-CTG-G
IL17A	Fw GGA-ACA-GAG-AGT-TAG-ACT-TGC-TG<br>Rev CTC-ATC-CTT-CAA-AGA-CAG-CCT-CA
MMP9	Fw ACT-CTA-CAC-CCA-GGA-CGG-C<br>Rev TCT-TGT-CGC-TGT-CAA-AGT-TCG
CXCR4	Fw TTG-TGG-GTG-GTT-TGT-GTT-CCA<br>Rev CTG-TGG-TCT-TGA-GGG-CCT-TG
TNFA (1)	Fw CCT-CTC-TCT-AAT-CAG-CCC-TCT-G<br>Rev GAG-GAC-CTG-GGA-GTA-GAT-GAG
TNFA (2)	Fw GCC-TGC-TGC-ACT-TTG-GAG-TG<br>Rev TCG-GGG-TTC-GAG-AAG-ATG-AT
INFG (1)	Fw TCG-GTA-ACT-GAC-TTG-AAT-GTC-CA<br>Rev TCG-CTT-CCC-TGT-TTT-AGC-TGC
INFG (2)	Fw CAG-CTC-TGC-ATC-GTT-TTG-GG<br>Rev CTG-TTT-TAG-CTG-CTG-GCG-AC
INOS	Fw TTC-AGT-ATC-ACA-ACC-TCA-GCA-AG<br>Rev TGG-ACC-TGC-AAG-TTA-AAA-TCC-C
MYD88	Fw GCA-GGA-GGA-GGC-TGA-GAA-GC<br>Rev CGG-ATC-ATC-TCC-TGC-ACA-AAC-T
ACTB	Fw GAT-CAT-TGC-TCC-TCC-TGA-GC<br>Rev ACT-CCT-GCT-TGC-TGA-TCC-AC

Table S2. Cytokines secreted by placentae according to maternal vaccination and infection status.

	Control	Vaccinated /not infected	Infected/not vaccinated	Infected/ vaccinated	Acute infected	*p*
IL-6	5716 ± 4862	6267 ± 3211	7343 ± 4550	6459 ± 4774	9199 ± 6229	0.4627
MCP-1	435.8 ± 268.5	623.5 ± 280.2	642.9 ± 494.4	675.8 ± 532.4	1211 ± 1320	0.1839
G-CSF	2462 ± 2384	6282 ± 3815	6937 ± 3881	4163 ± 4307	7647 ± 6289	0.0072

IFN-α2	4.6 ± 1.0	5.4 ± 0.2	5.4 ± 0.3	5.2 ± 0.4	5.7 ± 0.8	0.0012
IL-2	6.0 ± 1.1	7.1 ± 0.4	7.1 ± 0.6	6.7 ± 0.8	6.9 ± 1.0	0.0028
IFN-γ	9.0 ± 5.6	13.0 ± 6.4	14.1 ± 8.6	9.4 ± 8.0	16.7 ± 15.1	0.1068
IL-7	5.7 ± 301	7.8 ± 5.6	7.4 ± 3.7	6.8 ± 4.6	7.4 ± 3.7	0.6878
IL-1RA	1396 ± 988.0	2061 ± 1023	1977 ± 1399	2132 ± 1699	3264 ± 2012	0.0548
CXCL8	3125 ± 1704	4557 ± 1053	4521 ± 1189	3634 ± 1460	3894 ± 1907	0.0298
TNF-α	22.4 ± 18.0	9.0 ± 5.7	47.0 ± 77.2	38.4 ± 59.9	105.3 ± 100.7	<0.0001
IP-10	1224 ± 1463	983.4 ± 739.3	1294 ± 1224	1196 ± 1077	1984 ± 1395	0.1535
CCL3	41.9 ± 62.9	21.9 ± 17.0	38.6 ± 31.6	25.4 ± 29.0	55.1 ± 61.9	0.1552
IL-10	13.1 ± 6.0	16.9 ± 2.8	17.7 ± 5.0	14.5 ± 6.5	18.1 ± 8.7	0.0955

The mean ± SD values of the LegendPlex assay (in pg/ml) are shown, along with the results of the Kruskal–Wallis tests.
